# Phosphorus doped SnO_2_ thin films for transparent conducting oxide applications: synthesis, optoelectronic properties and computational models†
†Electronic supplementary information (ESI) available. See DOI: 10.1039/c8sc02152j


**DOI:** 10.1039/c8sc02152j

**Published:** 2018-08-23

**Authors:** Michael J. Powell, Benjamin A. D. Williamson, Song-Yi Baek, Joe Manzi, Dominic B. Potter, David O. Scanlon, Claire J. Carmalt

**Affiliations:** a Department of Chemistry , University College London , 20 Gordon Street , London WC1H 0AJ , UK . Email: c.j.carmalt@ucl.ac.uk; b Thomas Young Centre , University College London , Gower Street , London WC1E 6BT , UK; c Diamond Light Source Ltd , Diamond House, Harwell Science and Innovation Campus , Didcot , Oxfordshire OX11 0DE , UK

## Abstract

Phosphorus doped tin(iv) oxide (P:SnO_2_) films have been synthesised by an aerosol assisted chemical vapour deposition route. Triethyl phosphate was used as the phosphorus dopant source. The phosphorus concentration in solution was found to be key to electrical properties, with concentrations between 0.25–0.5 mol% phosphorus giving the lowest resistivities of the deposited films. The conductivity of the films synthesised improved on doping SnO_2_ with phosphorus, with resistivity values of 7.27 × 10^–4^ Ω cm and sheet resistance values of 18.2 Ω □^–1^ achieved for the most conductive films. Phosphorus doping up to 1.0 mol% was shown to improve visible light transmission of the deposited films. The phosphorus doping also had a significant effect on film morphology, with varying microstructures achieved. The films were characterised by X-ray diffraction, scanning electron microscopy, UV/vis spectroscopy, Hall effect measurements and X-ray photoelectron spectroscopy. The data generated was used to build computational models of phosphorus as a dopant for SnO_2_, showing that the phosphorus acts as a shallow one-electron n-type donor allowing for good conductivities. Phosphorus does not suffer from self-compensation issues associated with other dopants, such as fluorine.

## Abstract 2

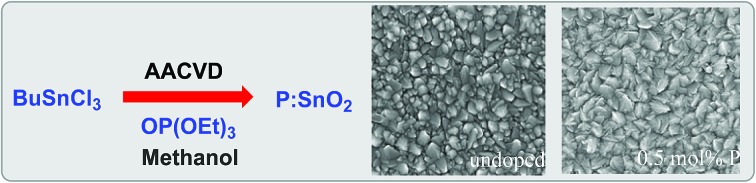
Phosphorus doped tin(iv) oxide (P:SnO_2_) films, with resistivity values of 7.27 × 10^–4^ Ω cm and improved visible light transmission, have been synthesised by AACVD.

## 1. Introduction

Transparent conducting oxides (TCOs) are fundamentally important in solar cells,^1^–^3^ flat-screen displays,^4^,^5^ organic light emitting diodes,^6^,^7^ touchscreen displays^8^,^9^ and liquid crystal displays.^10^ There are several techniques for depositing thin films of TCO materials, including, magnetron sputtering,^11^–^13^ spray pyrolysis,^14^,^15^ atmospheric pressure chemical vapour deposition,^16^,^17^ aerosol assisted chemical vapour deposition (AACVD)^18^,^19^ and sol–gel synthesis.^20^,^21^

For industrial production of TCO materials high throughput techniques, such as chemical vapour deposition and magnetron sputtering, are favoured for producing conformal coatings over large areas of substrate. Atmospheric pressure CVD can deliver high growth rates, with highly conformal coatings and are used commercially in the production of functional coatings, such as Pilkington Activ™. Doping of materials by atmospheric pressure CVD techniques is not a trivial matter. This is due to the incorporation of the dopant being controlled by the vapour pressure of the precursor, small changes in temperature or gas flow rate can lead to dramatic changes in incorporation of dopants into the host matrix.

Aerosol assisted CVD relies on the ability to produce an aerosol from a solution containing a suitable metal precursor. This eliminates the need for the precursor to be volatile, with the precursor only needing to be soluble in a suitable solvent, *i.e*. a solvent that can be atomised to generate a mist, for synthesis to be achieved.^22^ This allows for a far larger range of precursors to be used for the synthesis of functional thin film coatings. In a typical AACVD reaction, the aerosol is carried to the reaction chamber by a carrier gas, where it passes over a heated substrate resulting in nucleation, reaction and film growth. AACVD has several advantages for depositing materials, such as greater sustainability,^23^ a wider range of precursors, precise control over dopant concentrations and ease of co-doping materials.^22^ AACVD also has the potential to be a scalable process, although consideration of the choice of solvent would be critical, with water being the ideal solvent for sustainability, safety and reduction of unwanted waste products.^24^

Wide-bandgap (above 3.1 eV) semiconductor metal oxides, such as indium tin oxide (ITO),^25^–^27^ Al-doped zinc oxide (AZO)^28^–^30^ and F-doped tin oxide (FTO)^5^,^31^,^32^ are commonly employed for use as TCOs. ITO has been shown to have film resistivities as low as 5 × 10^–5^ Ω cm, mobilities of *ca*. 100 cm^2^ V^–1^ s^–1^ and charge carrier densities of 1.5 × 10^21^ cm^–3^.^33^ For AZO, film resistivities of 2 × 10^–4^ Ω cm, mobilities of *ca*. 50 cm^2^ V^–1^ s^–1^ and charge carrier densities of 5 × 10^20^ cm^–3^ have been reported.^34^ FTO has been shown to have film resistivities as low as 4 × 10^–4^ Ω cm, mobilities of *ca*. 40 cm^2^ V^–1^ s^–1^ and charge carrier densities of 4 × 10^20^ cm^–3^.^5^ ITO is the most commonly used TCO material in industry, owing to the low resistivity, high charge carrier concentration and high charge carrier mobility of the material.^35^ ITO, however, is expensive due to the high price of extracting and processing indium.^36^ There are also concerns over the relative scarcity of indium and the competing demands for indium from flat-screen displays and photovoltaic applications.^37^ As a consequence, there has been a focus on producing TCO materials that do not require the use of indium.

Tin(iv) oxide is an ideal candidate for use as a TCO material. Undoped SnO_2_ has a band-gap of ∼3.6 eV making it transparent to visible wavelengths,^38^ it is also intrinsically an n-type semiconductor and can be doped to improve its electrical properties. Common dopants to improve the electrical properties include; fluorine,^5^,^31^,^39^ antimony,^40^–^42^ tantalum^43^–^46^ and niobium.^46^–^48^ Although there have been limited reports on the use of phosphorus as a dopant to improve electrical properties,^49^–^53^ to the best of our knowledge, there has yet to be a report on the use of phosphorus doping of tin(iv) oxide synthesised by AACVD. Outlined in this paper is the effect that the phosphorus precursor, triethyl phosphate, has on the electrical, visible and morphological properties of SnO_2_ thin films deposited by AACVD. Coupled to the experimental work are computational studies that explore why phosphorus is an effective n-type dopant for SnO_2_ and showing how the phosphorus contributes to the improvement of the functional properties of the deposited films. Combining the experimental and computational results displays the potential of further increasing the functional properties of SnO_2_ thin films for TCO materials.

## 2. Experimental

All chemicals were used as bought, without further purification: monobutyltin trichloride (95%, Sigma-Aldrich), triethyl phosphate (99.8%, Sigma-Aldrich) and methanol (99.9%, Fisher), were used as the tin and phosphorus precursors and solvent respectively. Compressed air (21% (±0.5%) O_2_ in N_2_) was used as the carrier gas for all reactions, supplied from BOC. The glass substrate used for depositions was 3.2 mm thick plain float glass with a 50 nm thick SiO_2_ barrier layer (Pilkington/NSG).

### 2.1 Thin film synthesis

In a typical deposition, triethyl phosphate (0.0136 g, 7.5 mmol) was added to BuSnCl_3_ (0.3 g, 1.1 mmol) dissolved in methanol (20 mL) with stirring. The methanol solution was allowed to stir for *ca.* 10 min. Varying molar ratios of the P to Sn precursor were used to deposit a range of films. In the 7.5 mol% P:Sn film, the amount of triethyl phosphate to use (0.0136 g, 7.5 mmol) was calculated from the amount of BuSnCl_3_ in the solution. Precursor solutions containing 0.0, 0.1, 0.25, 0.5, 1, 1.5, 5 and 7.5 mol% P:Sn content were prepared in this manner.

The AACVD thin film depositions were carried out as detailed elsewhere.^31^ Briefly, a carbon block heater comprising the lower half of the reactor was used to maintain the substrate temperature using a k-type thermocouple. Depositions were carried out on Pilkington silica-coated barrier glass (50 nm SiO_2_ coated on one side of float glass) in order to prevent unwanted leaching of ions from the glass into the thin film.^54^ Prior to deposition, the glass substrates were cleaned with soapy water, isopropanol and acetone and were then left to air dry. The substrate was then loaded into the reaction chamber along with a second piece of float glass suspended 8 mm above (silica barrier layer pointing down) to ensure laminar flow during deposition. An aerosol mist of the precursor solution was generated using a ‘Liquifog’ piezo ultrasonic atomizer from Johnson Matthey, which uses an operating frequency of 1.6 MHz to produce a mode droplet size of *ca*. 3 μm. The mist was transported into the reactor *via* a baffle, using compressed air, as the carrier gas, at a constant flow-rate of 1.0 L min^–1^. The exhaust of the reactor was vented into a fume cupboard. When the precursor solution and associated aerosol mist had been completely emptied from the bubbler, the coated substrate was cooled to below 100 °C before being removed from the reactor. Deposition temperatures were fixed at 550 °C, as below this temperature carbon contamination made the films less visibly transparent. Typical deposition times were between 30–40 min. Sample descriptions can be found in Table 1.

**Table 1:** Descriptions for thin films deposited, their lattice cell parameters and volumes and P : Sn ratio from EDX. All samples were deposited at 550 °C with compressed air used as the carrier gas. The mol% of triethyl phosphate in precursor solution is given and the at% P in the resulting film as determined from EDX. Undoped lattice parameters and cell volume values were obtained from ICSD reference SnO_2_ (9163-ICSD)

Sample description mol% of triethyl phosphate in solution	Lattice parameter *a* = *b* (Å)	Lattice parameter *c* (Å)	Cell volume (Å^3^)	P : Sn ratio in film/at%
Undoped SnO_2_ thin film	4.738(1)	3.187(2)	71.53	—
0.1% P:SnO_2_ thin film	4.749(1)	3.181(1)	71.75(2)	—
0.25% P:SnO_2_ thin film	4.7444(5)	3.1905(6)	71.82(1)	—
0.5% P:SnO_2_ thin film	4.7449(4)	3.1880(7)	71.78(2)	0.26 : 99.74
1.0% P:SnO_2_ thin film	4.7419(1)	3.190(1)	71.70(3)	0.62 : 99.38
1.5% P:SnO_2_ thin film	4.737(1)	3.188(1)	71.53(3)	0.69 : 99.31
5.0% P:SnO_2_ thin film	4.740(2)	3.184(2)	71.55(4)	1.42 : 98.58
7.5% P:SnO_2_ thin film	4.745(2)	3.182(2)	71.65(5)	—

### 2.2 Thin film characterisation

Scanning electron microscope images were recorded on a Jeol JSM-6301F SEM at an acceleration voltage of 5 kV. X-ray diffraction (XRD) patterns were recorded using a Bruker D8 Discover X-ray diffractometer using monochromatic Cu K_α1_ and Cu K_α2_ radiation of wavelengths 1.54056 and 1.54439 Å respectively, emitted in an intensity ratio of 2 : 1 with a voltage of 40 kV and a current of 40 mA. The incident beam angle was 1° and data was collected between 5° and 66° 2*θ* with a step size of 0.05° at 1.0 s per step. All diffraction patterns obtained were compared with database standards (ICSD). Unit cell volumes and lattice parameters were calculated from the XRD data using GSAS and EXPGUI programs. X-ray photoelectron spectroscopy was conducted on a Thermo Scientific K-alpha spectrometer with monochromated Al Kα radiation, a dual beam charge compensation system and constant pass energy of 50 eV (spot size 400 μm). Survey scans were collected in the binding energy range 0–1200 eV. High-resolution peaks were used for the principal peaks of Sn (3d), O (1s), P (2s) and C (1s). Data was calibrated against C 1s (285.0 eV). Data was fitted using CASA XPS software. UV/vis spectra were recorded on a Perkin Elmer Lambda 950 UV/vis/NIR Spectrophotometer in both transmission and diffuse reflectance mode. A Labsphere reflectance standard was used as a reference for the UV/vis measurements. Room temperature Hall effect measurements were carried out on an Ecopia HMS-3000 set up in the Van der Pauw configuration. Measurements were taken using a 0.58 T permanent magnet and a current of 1 μA. Tests were carried out on square-cut samples measuring ≈1 × 1 cm. Silver paint (Agar Scientific) was used to form ohmic contacts, which were tested on the in-built software prior to measurement. The Hall effect method was used to find the *ρ*, *μ* and *n* using measured film thickness values as obtained from a Filmetrics F20 machine operating in reflectance mode in air against an as-supplied SnO_2_ standard.

### 2.3 Computational modelling

#### Theoretical methodology

The intrinsic and extrinsic defects simulated in this work were calculated using *ab initio* density functional theory (DFT) utilising the periodic code VASP.^55^–^58^ The projector-augmented wave (PAW)^59^ method was used to account for the interactions between the core and valence electrons for each species (Sn[Kr], O[He], P[Ne]). The geometric optimisations and electronic relaxations were carried out using the PBE0 (Perdew–Burke–Ernzerhoff) hybrid functional formalised by Adamo and Barone.^60^,^61^ PBE0 gives an accurate description of the band gap of SnO_2_ compared to standard DFT functionals which are limited by their failure to describe the self-interaction error. PBE0 has also been shown to accurately predict the properties of tin-based oxides.^62^–^71^ The 72 atom 2 × 2 × 3 supercells were based on a previously calculated geometry relaxation carried out on the conventional cell for SnO_2_ using a 400 eV plane-wave cutoff and a 4 × 4 × 6 Γ-centred *k*-point mesh.^62^ All the defect supercells and their respective charge states were calculated using Γ-centred *k*-point meshes of 2 × 2 × 2 and plane-wave energy cut-offs of 400 eV. Each structural optimization involved the relaxation of the ions whilst keeping the lattice vectors/angles/volumes fixed. Convergence was deemed complete when the forces on all the atoms were <0.01 eV atom^–1^. The limiting phase, P_2_O_5_ was relaxed using a planewave energy cut-off of 600 eV and a Γ-centred *k*-point mesh of 4 × 4 × 6.

#### Defect formalism

The corrected formation energy of a defect in charge state ‘*q*’, Δ*H*_f_(*D*,*q*) can be defined as:1
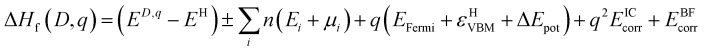

*E*^*D*,*q*^ refers to the total energy of the defective supercell in charge state ‘*q*’ and is in reference to the total energy of the host supercell, *E*^H^. *E*_*i*_ and *μ*_*i*_ correspond to the elemental reference energies (Sn_(s)_, O_2(g)_, P_(s)_) and their respective chemical potentials. The number of electrons added to or taken away from the external reservoir is notated by *n*. *E*_Fermi_ refers to the Fermi level and ranges from the valence band maximum (VBM) at 0 eV to 6 eV (∼2.4 eV above the conduction band minimum (CBM)). *ε*HVBM is the eigenvalue of the VBM of the host material. Due to the ‘finite’ size effects of the supercell, three corrections are applied. Firstly, a potential alignment term Δ*E*_pot_ must be added to correct the difference between the potential of the defective supercells and the host supercell. Secondly, an image-charge correction is applied, *E*ICcorr, which, due to the long ranged nature of the coulomb interaction^72^,^73^ corrects for the interaction of the charged defect and its own periodic images. The scheme used herein uses an image charge correction formalised by Hine and Murphy.^74^ Lastly, due to the high defect concentrations present in supercell calculations, a band filling term (*E*BFcorr) is applied to shallow defects in a method by Lany and Zunger.^75^,^76^

#### Thermodynamic limits

The growth conditions can be represented by the chemical potentials (*μ*_*i*_) and thus simulate the experimental partial pressures of preferential n and p-type defect formation. These are relative to the calculated enthalpy of SnO_2_:

2 *μ*_Sn_ + 2*μ*_O_ = Δ*H*SnOf^_2_^ = –5.27 eV (experiment = 5.98 eV (ref. 77))

Two growth conditions; Sn-rich/O-poor (n-type favourable) and Sn-poor/O-rich (p-type favourable) regimes can therefore be defined limited by the growth of Sn_(s)_ and O_2(g)_ respectively:

3 Sn-rich/O-poor conditions: *μ*_Sn_ = 0 eV; *μ*_O_ = –2.64 eV

4 Sn-poor/O-rich conditions: *μ*_Sn_ = –5.27 eV; *μ*_O_ = 0 eV

The solubilities of the phosphorus species are limited by the formation of a secondary phase, P_2_O_5_:

5 2*μ*_P_ + 5*μ*_O_ = Δ*H*Pf^_2_O_5_^ = –14.80 eV (experiment = –15.59 eV (ref. 78))

*μ*_P_ is therefore calculated to be –0.80 eV and –7.40 eV under Sn-rich/O-poor and Sn-poor/O-rich conditions respectively.

The thermodynamic transition levels (*q*/*q*′) can be calculated using the equation:6
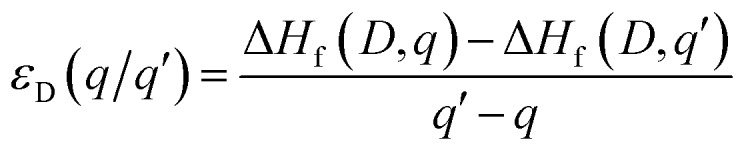



And show the evolution of a defect from charge state *q* to *q*′ at a certain Fermi level position. These are useful experimentally as they can be seen in techniques such as deep level transient spectroscopy (DLTS).

##### Dependence on oxygen partial pressure and temperature

In order to gain a snapshot of the defect chemistry under experimental conditions, the dependence of *μ*_O_ on the oxygen partial pressure and temperature can be determined using the equation:^79^7
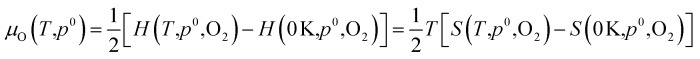

*T*, *H* and *S* are temperature, enthalpy and entropy respectively and *p*^0^ = 1 atm with reference to a zero state; 
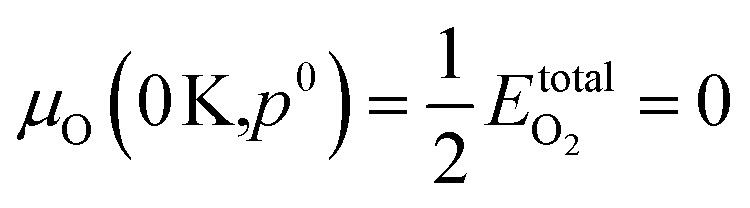
.^80^,^81^ The P:SnO_2_ films were carried out using AACVD at ∼900 K/1 atm allowing us to determine the oxygen chemical potential using data from thermochemical tables,^82^ therefore: *μ*_O_(*T*,*p*^0^) = –0.97 eV.

## 3. Results and discussion

### 3.1 Thin film characterisation

Thin films of P-doped SnO_2_ (P:SnO_2_) were synthesised from monobutyltin trichloride and triethyl phosphate by aerosol assisted chemical vapour deposition (AACVD) with compressed air as the carrier gas. The concentration of the phosphorus was varied in solution. All films were synthesised at 550 °C, with typical deposition times being 30–40 min.

**Fig. 1:**
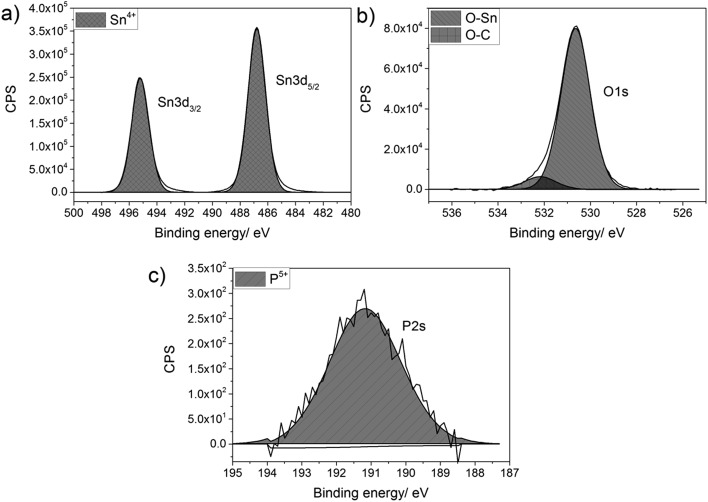
Typical X-ray photoelectron spectra for (a) Sn 3d environment, (b) O 1s environment and (c) P 2s environment for P-doped SnO_2_ films synthesised by AACVD at 550 °C.

The films were analysed for their elemental content *via* energy-dispersive X-ray spectroscopy (EDX) and X-ray photoelectron spectroscopy (XPS). XPS was also used to determine the oxidation state and environments for the elements present in the synthesised film, as shown in Fig. 1. In the XPS, the Sn 3d and P 2s environments were probed, since the Sn 3d and P 2p regions overlap and hence the P 2s had to be used to determine the oxidation state of the phosphorus. For all films deposited, there was only a single Sn environment, which gave values of 487.0 and 495.2 eV for the Sn 3d_5/2_ and Sn 3d_3/2_ respectively. These values matched with literature values for SnO_2_ (±0.2 eV).^83^,^84^ The oxygen environment gave a combination of O–Sn and O–C at 530.6 and 532.4 eV respectively (±0.2 eV), in agreement with literature values.^85^,^86^ The P 2s peak occurred at a value of 191.2 eV (±0.2 eV), which is commonly seen for phosphorus in the 5+ state.^87^,^88^ As the phosphorus precursor, triethyl phosphate, also formally has the phosphorus as P^5+^, this suggests that the phosphorus was not reduced when it was incorporated into the SnO_2_ lattice. This is not surprising, as the depositions were performed in an oxygen rich environment, which should keep the phosphorus in its maximum oxidation state.

Although phosphorus was detected within the XPS data, the signal was weak with a high noise to signal ratio, which indicates that the at% of phosphorus in all the P-doped samples were ∼1 at% or below, since this is the threshold of reliable detection for XPS. Therefore, although the phosphorus can be detected and can be seen in both the change in morphology from the SEM images and change in electrical resistivity from the Hall effect data (*vide infra*), the measured values cannot be included as the error on these would be larger than the value calculated from the XPS data.

To determine whether the phosphorus was segregated in the films, or uniformly dispersed, depth profiling was performed on the samples. This showed that the phosphorus was bulk segregated, with the P to Sn ratio increasing on etching of the films. In order to determine the amount of P present in each of the films EDX was used. The EDX results showed a gradual increase in the P content in the films with increasing mol% P precursor in the AACVD precursor solution (Table 1) from 0.5 mol% until 5 mol%. For films deposited using ≤0.25 mol% and 7.5 mol% triethyl phosphate, the P content in the films was too low to be accurately reported (<0.1 at%) although XPS and the observed change in morphology and XRD (*vide infra*) provide evidence of low quantities of P in these films. AACVD precursor solutions containing 0.5, 1.0 and 1.5 mol% triethyl phosphate produced SnO_2_ films with 0.26, 0.62 and 0.69 at% P, respectively. The films formed from 5 mol% triethyl phosphate in the precursor solution contained 1.42 at% before doping to very low levels using 7.5 mol%, suggesting possible saturation was reached at 5 mol% P. A similar effect was observed in the deposition of Sb-doped TiO_2_ films *via* AACVD where precursor solutions with >2.5 mol% Sb inhibited the incorporation of Sb in the TiO_2_ and reduction of the Sb(OEt)_3_ precursor occurred.^89^

**Fig. 2:**
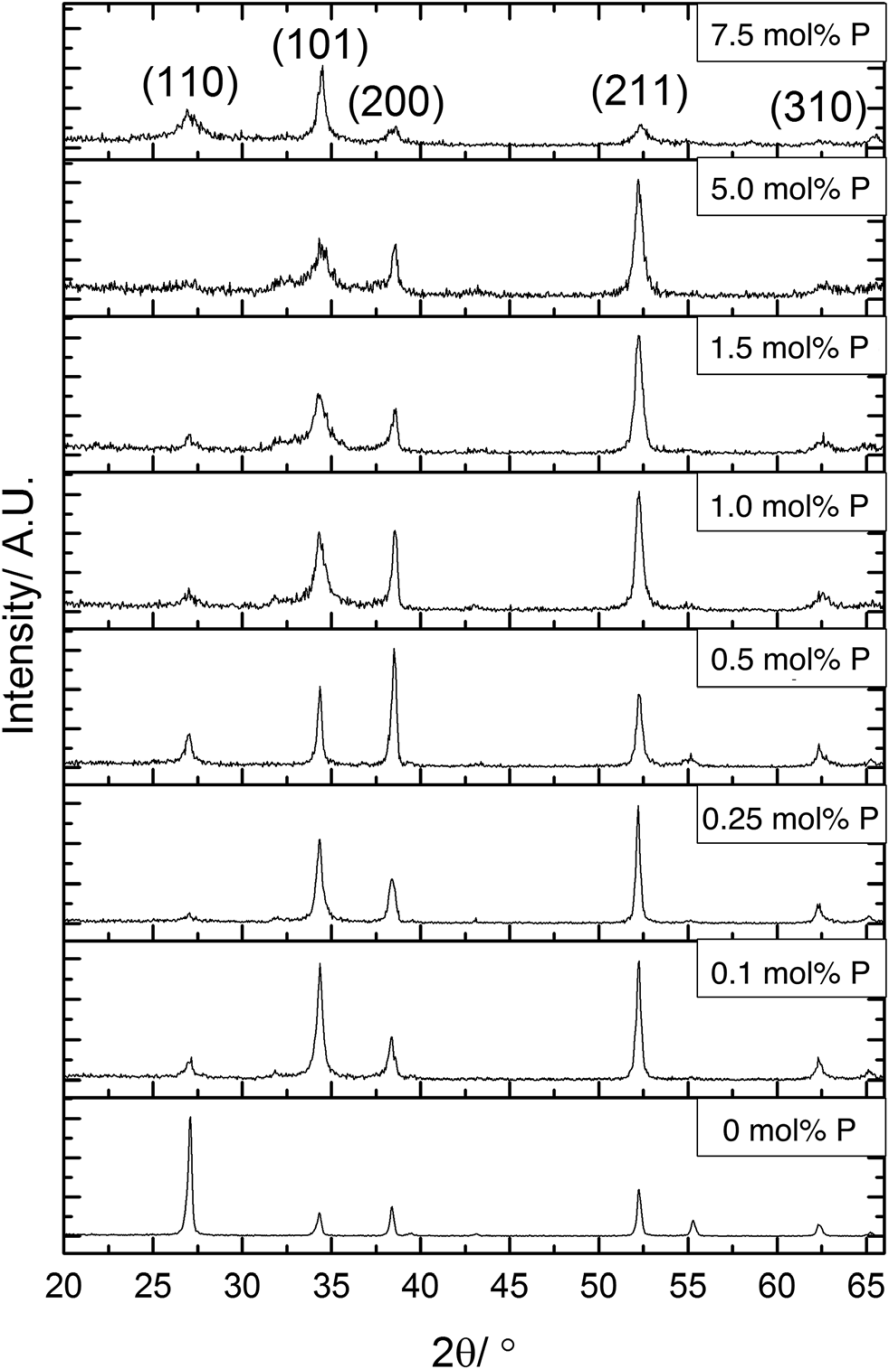
X-ray diffraction patterns for P:SnO_2_ thin films deposited by AACVD at 550 °C. A change in preferred orientation is observed between undoped, and inclusion of phosphorus into the crystal structure.

X-ray diffraction (XRD) was used to determine the phase present in the samples synthesised, Fig. 2. All XRD patterns displayed diffraction peaks that were referenced to the cassiterite phase of SnO_2_. The incorporation of phosphorus has several effects on the patterns displayed. The most obvious change was to the intensity of the (110) plane, this was the most intense diffraction peak for the undoped SnO_2_ film. On incorporation of phosphorus, however, the intensity of the reflection of this plane was significantly reduced in the XRD data. The intensity of this diffraction peak then remains low throughout the various phosphorus dopant concentrations. This suggests that phosphorus retards crystal growth in this direction.

Preferred orientation was observed in (101) and (211) planes for films formed using ≤0.25 mol% triethyl phosphate. The (200) plane was suppressed when using 1.5 mol% or higher P precursor, with preferred orientation occurring in the (101)/(200)/(211) planes for films formed with 0.5 mol% and 1.0 mol% triethyl phosphate. The highest amount of 7.5 mol% P precursor showed that the (211) plane was also suppressed. Preferred orientation in the (211) plane has been observed previously for Sb-doped SnO_2_ films.^90^,^91^ For F-doped SnO_2_ thin films, a preferential orientation towards the (200) is commonly observed for highly conductive films.^5^,^92^

It was noticeable that at high dopant concentrations, the phosphorus appeared to reduce the overall crystallinity of the films since all the diffraction peaks become weaker and less well defined. Interestingly, this effect was only displayed for the (211) plane at concentrations above 5 mol% triethyl phosphate in the precursor solution, whereas the (101) and (200) display this reduction in intensity at lower concentrations of phosphorus. This has been previously observed for phosphorus doped SnO_2_ films synthesised by the simultaneous oxidation of phosphine and tetramethyltin,^49^ where above 3.2 at% phosphorus content, the crystallinity of the films rapidly diminished.

From the XRD patterns a shifting of the diffraction peaks to higher angles at greater phosphorus concentrations was observed. This suggests that phosphorus was being incorporated into the SnO_2_ lattice, with the smaller ionic radius of P(v) (34 pm compared to Sn(iv) which is 71 pm) leading to a reduction in the lattice parameters.

The data from the XRD patterns was used to calculate the lattice parameters for the films and observe how these were affected by the inclusion of phosphorus, as shown in Table 1. Due to the poor crystallinity of the films at higher dopant concentrations, there was a higher error in the calculated unit cell volumes. The trend apparent in the data showed that phosphorus incorporation leads to a reduction of the crystal lattice. Although there is an apparent initial increase in lattice parameters, this is within the error for the data quality and so is most likely an artefact of the model used to calculate the lattice parameter data. As the phosphorus in the precursor is in the 5+ state, it will most likely replace on tin sites. With phosphorus being a smaller atom than tin, this would lead to a reduction in lattice parameters, which is seen for higher doping levels.

**Fig. 3:**
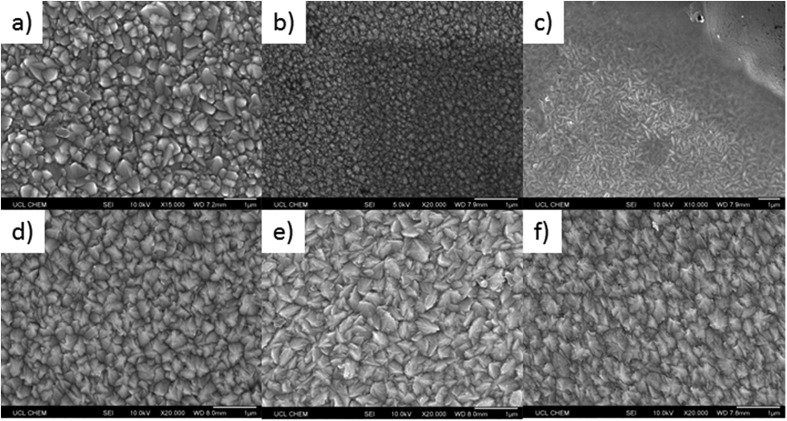
Typical SEM images showing the surface morphology for (a) undoped SnO_2_, (b) 0.1 mol% P:SnO_2_, (c) 0.25 mol% P:SnO_2_, (d) 0.5 mol% P:SnO_2_, (e) 1.0 mol% P:SO_2_, and (f) 1.5 mol% P:SnO_2_ thin films on glass. All samples were produced at 550 °C, by aerosol assisted CVD using compressed air as the carrier gas.

To determine the effect of phosphorus doping on the morphology of the deposited SnO_2_ films, scanning electron microscopy (SEM) images were obtained for all samples, Fig. 3. As with the XRD patterns, Fig. 2, the incorporation of phosphorus had a large effect, such that a variation in surface morphology of the deposited samples was observed. Undoped SnO_2_, Fig. 3a, displayed typical pyramidal structures associated with SnO_2_ deposited by AACVD processes.^93^

Phosphorus doping has a remarkable effect on the morphology of the samples. Even at low %mol in solution (0.1 mol%), Fig. 3b, a lowering of the average size of the particles present was observed. The particles are also much rounder, with a loss of the pyramidal shape. With higher phosphorus %mol in the precursor solution, the particles become more rod-like in shape. This is supported in the XRD patterns for this sample (0.25 mol%), where the (101) plane had high intensity and the (110) plane was very low in intensity, this is known to favour rod-like particle formation for SnO_2_.^94^ Interestingly, although films formed using 0.1 mol% and 0.25 mol% P precursor in solution show similar XRD patterns and relative preferred orientations, the 0.1 mol% has a higher relative intensity in the (110) plane, which appears to prevent the growth of the rod-like particles.

The other feature in the morphology for films deposited using 0.25 mol% triethyl phosphate in solution (Fig. 3c) is the presence of a continuous layer that covers the rod-like particles; this is shown in the top right hand corner of the image. This uniform layer does not appear to be made of any obvious individual particles, which suggests that phosphorus incorporation can control grain boundary growth. Control of grain boundaries has been shown to improve the properties of graphene,^95^ silicon^96^ and zirconia^97^ layers/thin films.

With increasing phosphorus %mol in the starting precursor solution, the pyramidal particle shape was once again present. The particle shapes were not, however, identical to undoped SnO_2_. The particles were more angular and jagged, resembling shark's teeth, Fig. 3d–f. This coincides with a loss in intensity in the (101) plane for the XRD patterns for these samples and a change in preferential orientation to the (200) plane for films deposited using 0.5 mol% P precursor. This type of morphology has been previously observed for F-doped SnO_2_ with preferred orientation in the (200) plane.^98^

**Fig. 4:**
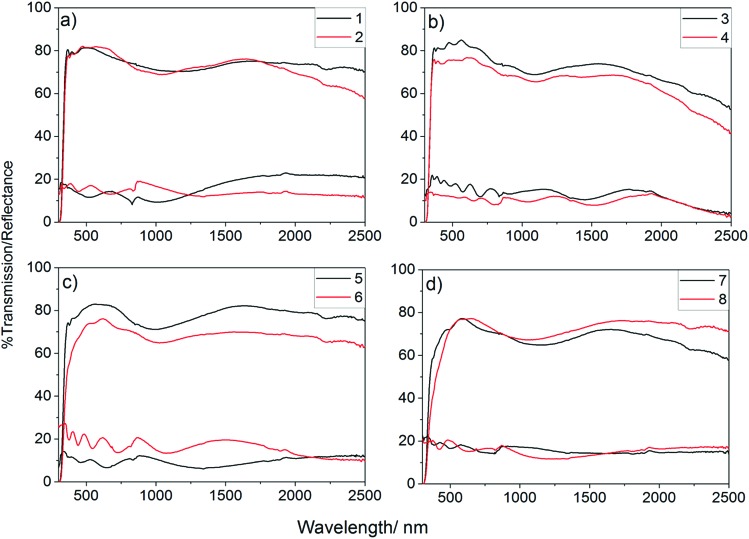
UV/vis/NIR spectra for P-doped SnO_2_ films; (a) undoped SnO_2_ (1) and 0.1 mol% P:SnO_2_ (2); (b) 0.25 mol% P:SnO_2_ (3) and 0.5 mol% P:SnO_2_ (4); (c) 1 mol% P:SnO_2_ (5) and 1.5 mol% P:SnO_2_ (6); and (d) 5 mol% P:SnO_2_ (7) and 7.5 mol% P:SnO_2_ (8). In all cases the films showed high transmission (*ca*. 80%) and low reflection (<20%). All films deposited by AACVD at 550 °C using air as carrier gas.

UV/vis spectra were obtained for all deposited films to determine the effect of the incorporation of phosphorus on the optical properties of the films, Fig. 4. As shown, the phosphorus incorporation initially led to a small increase in the visible light transmission of the films formed using 0.25 mol% triethyl phosphate in the precursor solution which gave visible light transmission *ca*. 82% (400–700 nm), this was above the visible light transmission of the undoped sample (80.8%). Further increasing the concentration, however, led to a significant worsening of the visible light transmission. Films that were deposited from solutions with higher than 1 mol% triethyl phosphate had a significant yellow hue to their colour, when observed in transmission, so it is not surprising that these films were poorer at allowing visible light to be transmitted. Colour centres are known to be caused by defects, such as oxygen vacancies, with doping of metal oxides often leading to an increase in the number of these defects.^99^,^100^

### 3.2 Functional testing

**Fig. 5:**
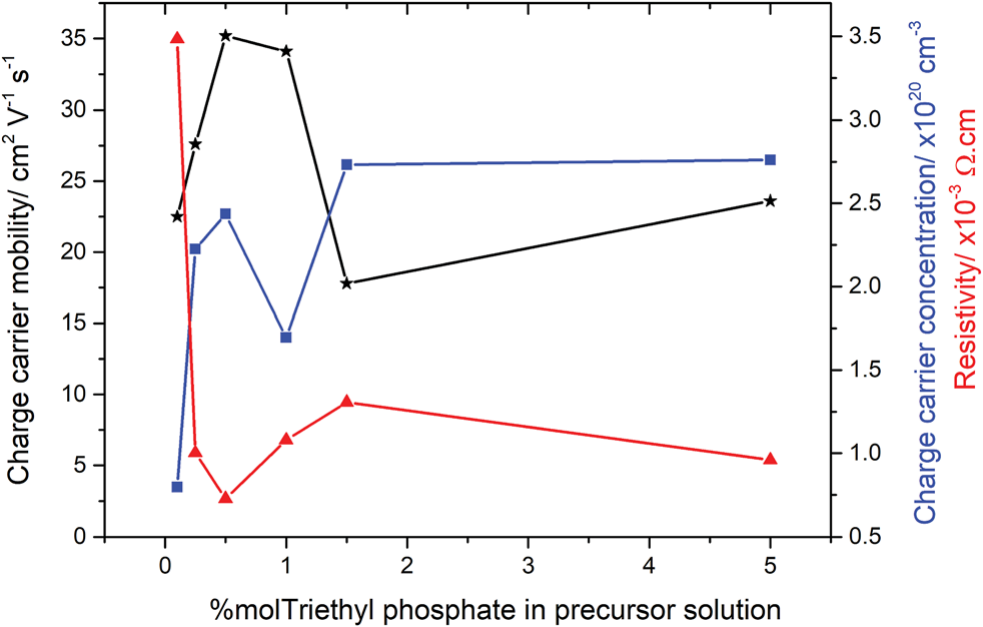
Electrical properties for the P-doped SnO_2_ films as obtained by Hall effect measurements.

In order to determine the electrical properties of the deposited films, Hall effect measurements were obtained, shown in Table 2. Undoped SnO_2_ and the use of high concentrations of triethy phosphate (7.5 mol% P) led to films that were too resistive for the Hall effect to measure the electrical properties. Increasing the amount of phosphorus doping (0.26–1.42 at% P in the films from 0.5–5 mol% triethyl phosphate precursor), however, had a dramatic impact on the resistivity values, as shown in Fig. 5. There was a clear trend observed in the data, where by increasing the phosphorus level from 0.1 to 0.5 mol% in solution led to films with improved electrical properties. The reduction in resistivity was accompanied by an increase in carrier density and mobility. At higher phosphorus doping, the electrical properties deteriorated, such that a slight increase in resistivity and reductions in charge carrier densities and mobilities were observed (films formed from 1.0 and 1.5 mol% triethyl phosphorus). Interestingly, the values recovered again around 5 mol% phosphorus in solution, before again becoming highly resistive above 7 mol%. The lowest resistivity values were for the films that were formed using 0.5 mol% triethyl phosphorus resulting in 0.26 at% P in the films. These films had resistivity values of 7.27 × 10^–4^ Ω cm, sheet resistance of 18.2 Ω □^–1^ and charge carrier mobility of 35.2 μ cm^–2^ V^1^ s^1^, these values are comparable to industry standards for F-doped SnO_2_ thin films, as shown in Table 2.

**Table 2:** Table of electrical and optical data for all P-doped SnO_2_ samples synthesised in air by AACVD of ^*n*^BuSnCl_3_ and OP(OEt)_3_ in methanol at 550 °C. Undoped SnO_2_ and the film from 7.5 mol% P were too resistive to obtain Hall effect measurements. Samples are compared to industrial F-doped SnO_2_ standards^*a*^

Sample	*d* μm^–1^	*n*/×10^20^ cm^–3^	*μ*/cm^2^ V^–1^ s^–1^	*ρ*/×10^–3^ Ω cm	*R*_Sh_/Ω □^–1^	*T*_*λ*550_/%	*T*_λ400–700_/%
Undoped SnO_2_	0.40	N/A	N/A	N/A	N/A	80.9	80.8
0.1 mol%	0.40	0.80	22.5	3.48	87.1	81.6	82.2
0.25 mol%	0.40	2.23	27.6	1.00	25.0	84.5	83.8
0.5 mol%	0.40	2.44	35.2	0.73	18.2	76.7	79.3
1.0 mol%	0.40	1.70	34.1	1.08	27.0	82.8	82.4
1.5 mol%	0.40	2.73	17.8	1.31	32.7	74.0	73.4
5.0 mol%	0.40	2.76	23.6	0.96	24.0	76.0	74.4
7.5 mol%	0.40	N/A	N/A	N/A	N/A	76.1	72.6

**Commercial standards**							
TEC™8	0.65	5.3	28	0.52	8.0	83	82
TEC™15	0.35	5.6	21	0.53	15.1	85	83
Asahi U™	0.90	2.2	32	0.88	9.8		

^*a*^ *d*: film thickness (±0.02 μm); *n*: charge carrier concentration; *μ*: charge carrier mobility; *ρ*: bulk resistivity; *R*_sh_: sheet resistance; *T*_*λ*550_; transmittance at 550 nm; *T*_*λ*400–700_: average transmittance over visible light range, 400–700 nm.

Interestingly, the most conductive film (from 0.5 mol% triethyl phosphate) displayed preferential orientation in the (200) plane, which is commonly seen for highly conductive SnO_2_ films.^101^,^102^ However, the other films formed from 0.25, 1.0, 1.5 and 5 mol% P precursor, display preferred orientation in the (211) plane but were still highly conductive. This suggests that the origin of the increased electrical conductivity is not the product of a particular crystallographic orientation, but a mix of morphological and electronic contributions.

The most surprising value about the doping of these films is the low level of phosphorus required to achieve improvements in electrical properties. Even using only 0.1 mol% P precursor resulted in a significant increase in the electrical conductivity of the deposited films. Conversely increasing phosphorus concentrations above 1.5 mol%, the electrical properties deteriorated. This was accompanied by the films becoming highly coloured, with a yellow hue obvious in the deposited films. This suggests that the phosphorus forms colour centres when in higher concentrations, with these colour centres also acting as places where either recombination or deflection of the electrons can occur.^103^ Although F-doping of SnO_2_ also requires low levels of incorporation, between 0.5–1 at%, phosphorus doping by AACVD requires even smaller levels of incorporation into the lattice.

Using the Hall effect data it was also possible to establish the dominant scattering mechanism for the films. This was achieved by plotting the mobility values against the charge carrier concentrations. For all the samples synthesised for this study, the mobilities were relatively low (17–35 cm^2^ V^–1^ s^–1^), whilst the charge carrier concentrations were relatively high (0.8–2.7 × 10^20^ cm^–3^), this suggests that the dominant scattering mechanism for all samples is due to grain boundary scattering. This type of scattering mechanism is typical of polycrystalline SnO_2_ thin films.^104^

These results show that phosphorus is an effective dopant for the synthesis of highly conductive n-type TCO thin films. Phosphorus doping also has a large impact on the preferred orientation and morphology of the films deposited, with low levels of phosphorus leading to round and rod-like particles with the development of pyramidal structures being observed at higher levels of doping, *ca*. 0.5 mol% triethyl phosphate in the precursor solution. Rod-like structures of SnO_2_ have been used for gas sensing applications,^105^,^106^ so being able to select for this kind of morphology by incorporation of a dopant is of potential interest.

Furthermore, phosphorus doping of SnO_2_ opens up a new direction for the development of TCO materials synthesised by aerosol assisted CVD. Although the electrical properties of these films were not as high as for the best quoted values for F-doped SnO_2_, they are still comparable to industry standards. The phosphorus dopant, triethyl phosphate has low toxicity, whereas many of the fluorine precursors are highly toxic. This makes the handling, storage and disposal of the phosphorus precursor easier, cheaper and safer than for common fluorine precursors, such as ammonium fluoride.

### 3.3 Computational modelling and results

The experimental work described above indicates that phosphorus is an effective n-type dopant for SnO_2_ and functional properties required for TCOs can be achieved. In order to explore how the phosphorus contributes to the improvement of the functional properties of the deposited films computational models of phosphorus as a dopant for SnO_2_ have been carried out. Phosphorus could potentially act both as an acceptor and donor dopant substituting on both O and Sn sites, as well as incorporating interstitially. Hence, theoretical calculations have been carried out on each of these defect environments as well as for the dominant intrinsic donor (V_O_) and acceptor (V_Sn_) defects.

**Fig. 6:**
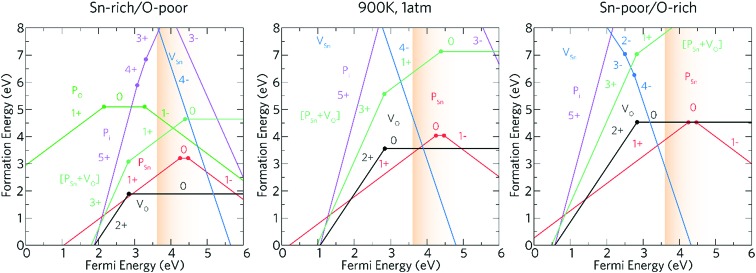
The charge transition level diagrams for Sn-rich/O-poor (left), 900 K, 1 atm (middle) and Sn-poor/O-rich (right). The Fermi energy goes from the VBM (0 eV) to ∼2.4 eV above the CBM (which is at 3.6 eV). The conduction band region is depicted by the graded orange area.

The transition level diagrams under Sn-rich/O-poor, 900 K, 1 atm and Sn-poor/O-rich conditions are shown in Fig. 6.

Oxygen vacancies, V_O_, and tin vacancies, V_Sn_, act as the two lowest energy intrinsic donor and acceptor defects respectively in SnO_2_. V_O_ is a negative-U defect and a deep donor with the 2+/0 transition level occurring ∼0.76 eV below the conduction band minimum (CBM) and as such, does not contribute significantly to the conductivity. This behaviour is in keeping with other theoretical^69^,^107^,^108^ and experimental^109^,^110^ results. V_O_ is a negative-U defect in other TCOs, such as ZnO,^69^,^111^–^113^ In_2_O_3_ (ref. 69 and 114–116) and BaSnO_3_ (ref. 67) and has been identified *via* positron annihilation spectroscopy.^117^ The neutral charge state of V_Sn_ occurs very high in energy under all growth regimes (under favourable Sn-poor/O-rich conditions, the formation energy is ∼8.37 eV) acting as a deep acceptor with the 0/1– transition level occurring ∼1.75 eV above the VBM.

#### P_Sn_

**Fig. 7:**
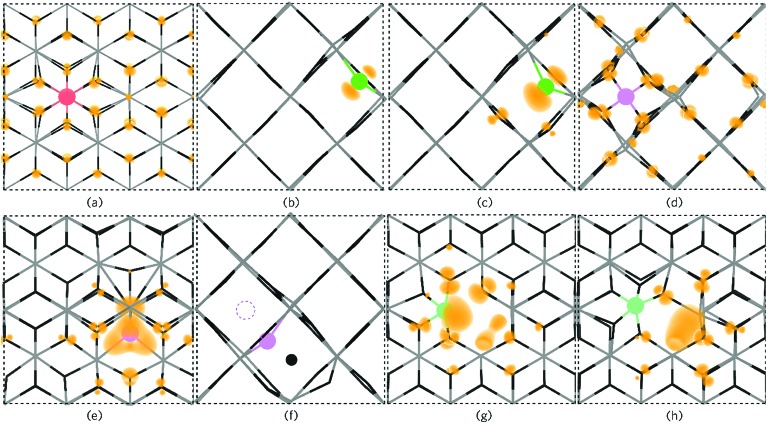
The partial electron charge densities (orange) for (a) P_Sn_ viewed along {010} with a charge density of 0.002 eV A^–1^, (b) P0O and (c) P_O_^1–^ as viewed along the {001} direction with a charge density of 0.02 eV A^–1^. P_i_^3+^ is depicted in (d) along the {001} direction with a charge density of 0.008 eV A^–1^ and P_i_^3–^ is shown in (e) along {010} with a charge density of 0.02 eV A^–1^ where a large triple polaron is seen. In (f), P_i_^3–^ is viewed along the {001} axis presenting the displacement of a lattice O to an interstitial site by P (the original interstitial position is shown by the dashed circle). (g) and (h) displays the defect cluster [P_Sn_ + V_O_] in both the neutral and 1+ charge states respectively as viewed along {010} and plotted from 0–0.01 eV A^–1^. In each panel the SnO_2_ is depicted by the grey (Sn) and black (O) wire frame model with the P species colour coded to Fig. 6.

Under all three growth regimes P_Sn_ is the dominant donor P defect and is a shallow donor. Under Sn-rich/O-poor conditions, the formation energy of P0Sn is ∼3.21 eV and rises to ∼4.53 eV under Sn-poor/O-rich conditions making P_Sn_ a relatively high formation energy donor. This indicates that incorporation of P under equilibrium conditions could be difficult. However, non-equilibrium approaches such as sputtering or MBE (molecular beam epitaxy) might be a relatively facile way to achieve higher levels of doping. Large lattice relaxations could rationalise this due to the significantly smaller phosphorus ionic radii to Sn (∼50% reduction in radii).^118^ Although P does not shift from the original Sn position (through all charge states) a reduction of ∼14% is seen for the P–O bond lengths (compared to Sn–O) which is commensurate with calculations carried out by Varley *et al.*^119^ Varley *et al.* also noticed that P_Sn_ acts a shallow donor with the electron occupying a conduction-band-like state. Work by Lany and co-workers have shown using HSE06 (Heyd–Scuzeria–Ernzerhoff) combined with GW (green's function) that P_Sn_ is a negative-U defect and that the 1+/1– transition level occurs ∼0.4 eV and ∼0.9 eV above the CBM for HSE06 and HSE06+GW respectively.^120^ In our calculations, however, both the 1+/0 and 0/1– transition levels are seen ∼0.65 and ∼0.87 eV above the CBM respectively. Our work also shows that in the 1– charge state, P_Sn_ delocalises some of the extra charge and is therefore not a true acceptor state, this is shown in Fig. 7a. V_Sn_^4–^ becomes the dominant compensating defect ∼1.37 eV above the CBM under Sn-rich/O-poor conditions and under Sn-poor/O-rich conditions V_Sn_^4–^ crosses P_Sn_^1+^ at ∼0.2 eV below the CBM. At 900 K, 1 atm compensation occurs at ∼0.3 eV above the CBM, which is consistent with degenerate n-type conductivity.

#### P_O_

P_O_ is an amphoteric defect, acting as both a deep donor and a deep acceptor defect with the 1+/0 transition level occurring ∼1.45 eV below the CBM and the 0/1– charge state occurs ∼3.27 eV above the VBM which is in agreement with previous HSE06 studies.^119^ P_O_ typically occurs relatively high in energy (∼5 eV under Sn-rich/O-poor conditions), most likely due to the relatively larger size of P to O. When incorporated, P shifts from the O site by around 3% in the *a* and *b* directions and a further 4% for the 1– charge state (which can be attributed to the increased electron localisation), this is shown in Fig. 7b and c for the neutral and 1– charge states respectively.

#### P_i_

Interstitial P (P_i_), whilst a shallow three electron donor, remains very high in energy under all growth conditions, under Sn-rich/O-poor conditions the formation energy of P0i is ∼9.3 eV. P_i_ is the dominant phosphorus defect at Fermi levels ∼1.9 eV above the VBM until the formation of P_Sn_ becomes more favourable (∼2.1 eV above the VBM). Under more O-rich environments the P_i_^5+^/P_Sn_^1+^ crossing point is pushed towards Fermi energies near the VBM. Being an amphoteric defect, P_i_ also acts as an acceptor, but any self-compensation occurs well above the conduction band minimum under all growth conditions. Large lattice distortions are seen for P_i_ in the 5+, 4+, and 3+ allowing P to assume a PO_4_ tetrahedral coordination, which has the effect of localising some of the electron charge on the adjacent Sn atoms. An illustration of this distortion for the 3+ charge state is shown in Fig. 7d, where the delocalised electron charge is seen. In the 3– charge state, P displaces an adjacent oxygen to the interstitial position (Fig. 7f), and a highly localised triple polaron forms on the P (Fig. 7e), however this charge state is highly unfavourable.

#### [P_Sn_ + V_O_]

Extended X-ray absorption fine structure (EXAFS) experiments on Sb-doped SnO_2_ have proposed the clustering of substitutional Sb with oxygen vacancies, which has also been corroborated through theoretical calculations.^121^,^124^ A similar analysis can be carried out in P-doped SnO_2_ by assessing the viability of such a cluster by calculating [Sb_Sn_ + V_O_] in both a ‘near’ (neighbouring each other) and ‘far’ (∼8 Å apart) configuration. From our calculations, we find that the ‘near’ configuration is more favourable than the ‘far’ configuration by ∼0.14 eV. A binding energy (*E*_BE_) of 0.45 eV was calculated using:*E*_BE_ = *E*^[Sb_Sn_+V_O_]^ + *E*^host^ – *E*^Sb_Sn_^ – *E*^V_O_^

Under Sn-rich/O-poor, 900 K, 1 atm and Sn-poor/O-rich growth conditions, the neutral charge state of [P_Sn_ + V_O_] is 4.64 eV, 7.13 eV and 8.59 eV respectively.^121^,^122^ It is likely, therefore, that this defect complex will form in negligible quantities or possibly form at high doping concentrations. [P_Sn_ + V_O_] acts as a shallow one-electron donor in SnO_2_ with the 1+/0 transition level occurring around 0.74 eV above the CBM. A further transition level (3+/1+) occurs in the band gap at 0.82 eV below the CBM. Fig. 7g and h show the partial charge electron density for [P_Sn_ + V_O_] in both the neutral (Fig. 7g) and 1+ (Fig. 7h) charge states. In the neutral charge state, the electron density can be seen to localise on the P and in the oxygen vacancy. In the 1+ charge state, an electron has been removed from the P atom leaving two electrons in the oxygen vacancy. It is therefore reasonable to assume that phosphorus remains in the 5+ oxidation state and that the formation of this defect complex exists due to lattice strain.

Based on the above results, our calculations show that P:SnO_2_ is a shallow one-electron n-type donor allowing for good conductivities. Phosphorus does not suffer from self-compensation issues like F:SnO_2_,^123^ however, towards higher doping concentrations the incorporation of P may need a non-equilibrium deposition technique due to the increase in formation energy. The computational calculations agree with the experimental observations, such that phosphorus was incorporated as P^5+^ (from XPS), and the conductivity of the films synthesised improved on doping SnO_2_ with phosphorus, with resistivity values of 7.27 × 10^–4^ Ω cm and sheet resistance values of 18.2 Ω □^–1^ achieved for the most conductive films, as expected for a one-electron n-type dopant. Moreover, only low dopant levels of P in the SnO_2_ were achieved, which correlate with the theoretical results indicating that incorporation of P under equilibrium conditions could be difficult. Combining the experimental results with computational modelling allowed for the determination of the effectiveness of phosphorus as a dopant for SnO_2_.

## 4. Conclusions

P-doped SnO_2_ thin films with excellent optical and electrical properties were synthesised by aerosol assisted chemical vapour deposition. The best performing films had a sheet resistance of 18.2 Ω □^–1^, charge carrier mobility of 35.2 μ cm^–2^ V^1^ s^1^ and resistivity of 7.27 × 10^–4^ Ω cm^–1^ which is comparable to industry standards. The phosphorus was shown to have a significant impact on the preferential orientation, morphology and crystallinity of the films, with a range of different morphologies observed in the microstructure of the polycrystalline films. Combining the experimental results with computational modelling allowed the determination of the effectiveness of phosphorus as a dopant for SnO_2_. Phosphorus has been shown to be a shallow level n-type dopant with the electrons possessing excellent mobility. This synthetic route opens up the possibility of using a common element to dope SnO_2_ films for transparent conducting oxide applications.

## Author contributions

S. B., M. J. P., D. B. P. and J. M. contributed experimental and characterisation techniques, B. A. D. W. contributed the computational modelling, D. O. S. and C. J. C. supervised the work, all authors contributed to writing the manuscript.

## Conflicts of interest

The authors declare no conflict of interest.

## Supplementary Material

Supplementary information
