# Supplementary figures and images for: Phosphorus doped SnO2 thin films for transparent conducting oxide applications: synthesis, optoelectronic properties and computational models
Source: Chem Sci. 2018 Aug 23;9(41):7968–80. doi: 10.1039/c8sc02152j (PMC6237145; doi:10.1039/c8sc02152j)

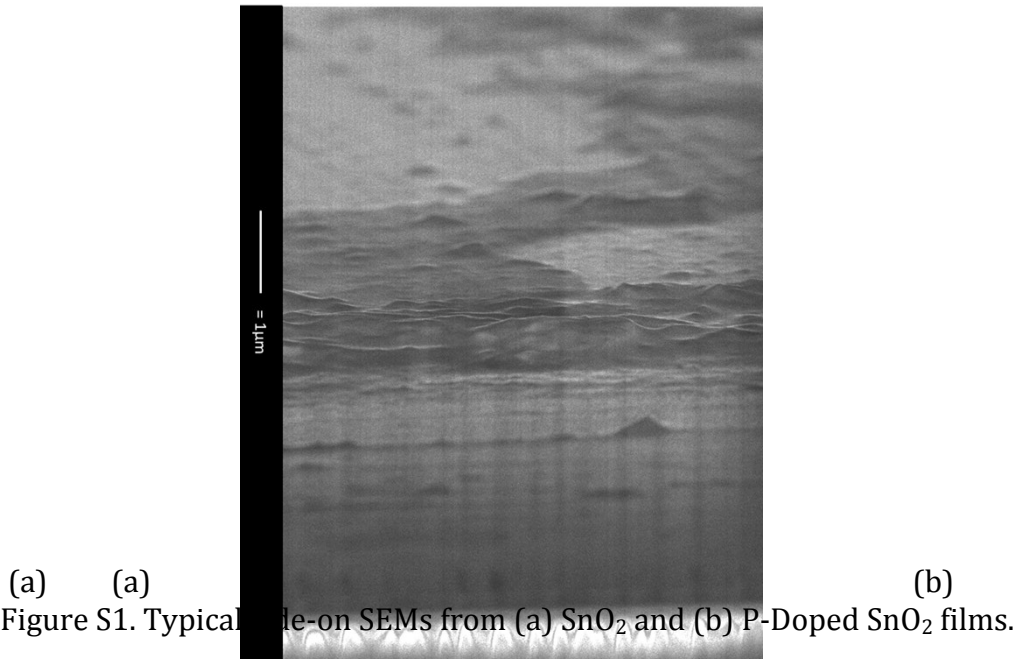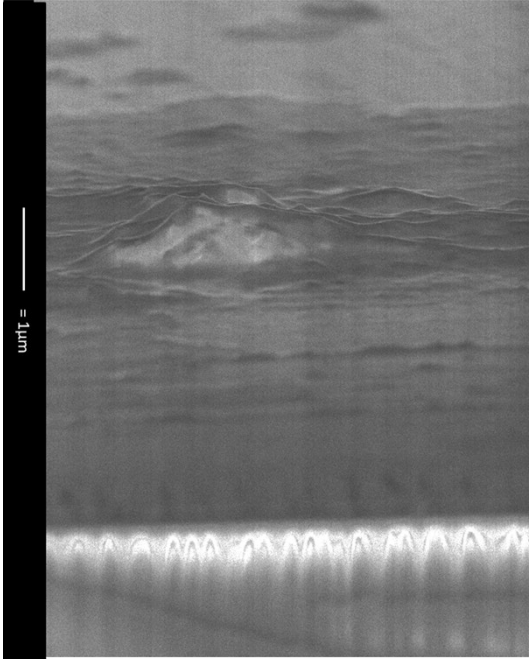

Supplement: Supplementary file 1 [file SC-009-C8SC02152J-s001.pdf]
